# Supplementary figures and images for: Alterations in the Gut Microbiome in the Progression of Cirrhosis to Hepatocellular Carcinoma
Source: mSystems. 2020 Jun 16;5(3):e00153-20. doi: 10.1128/mSystems.00153-20 (PMC7300357; doi:10.1128/mSystems.00153-20)

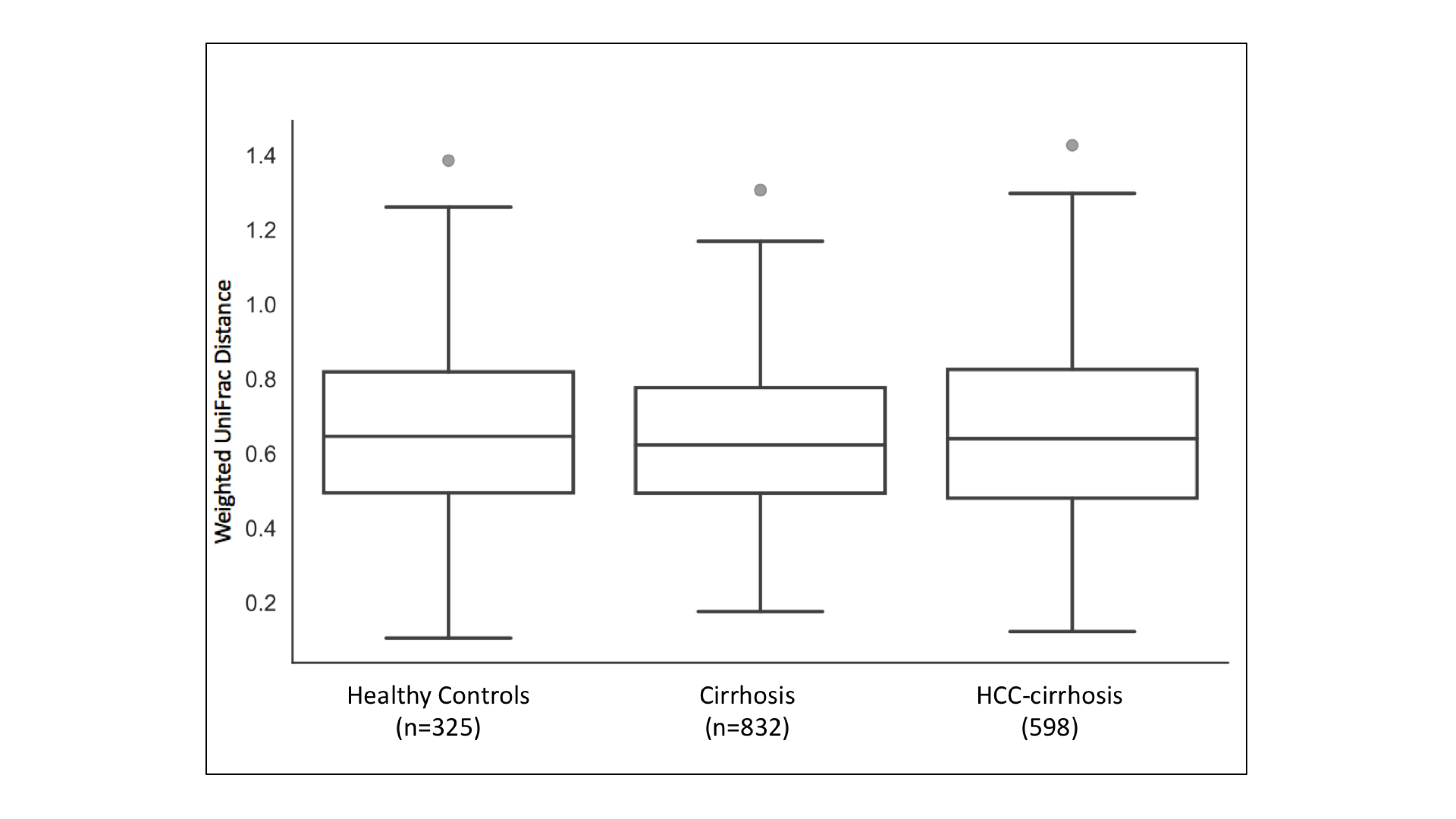

Supplement: FIG S1 [file mSystems.00153-20-sf001.tif]

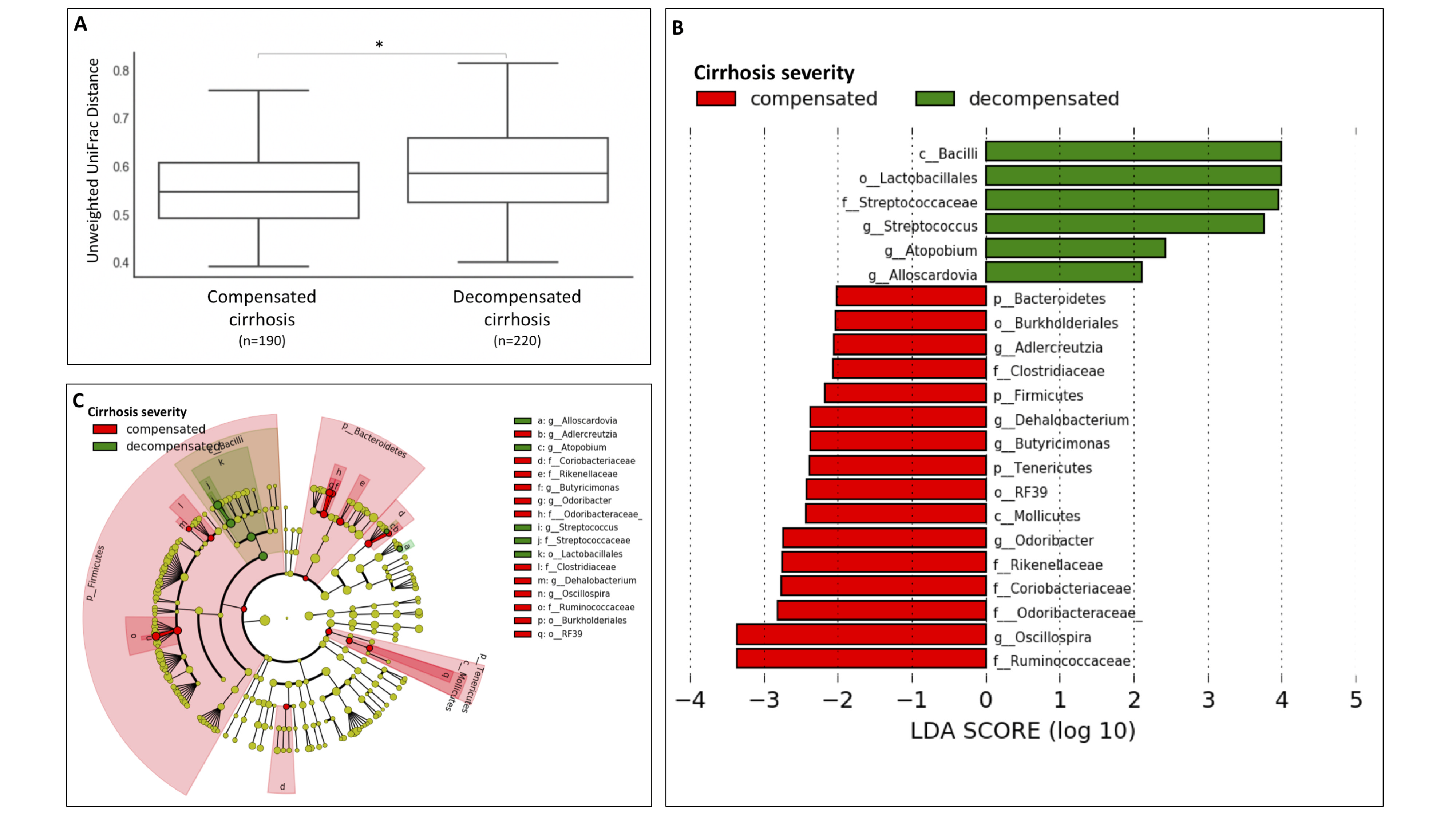

Supplement: FIG S2 [file mSystems.00153-20-sf002.tif]

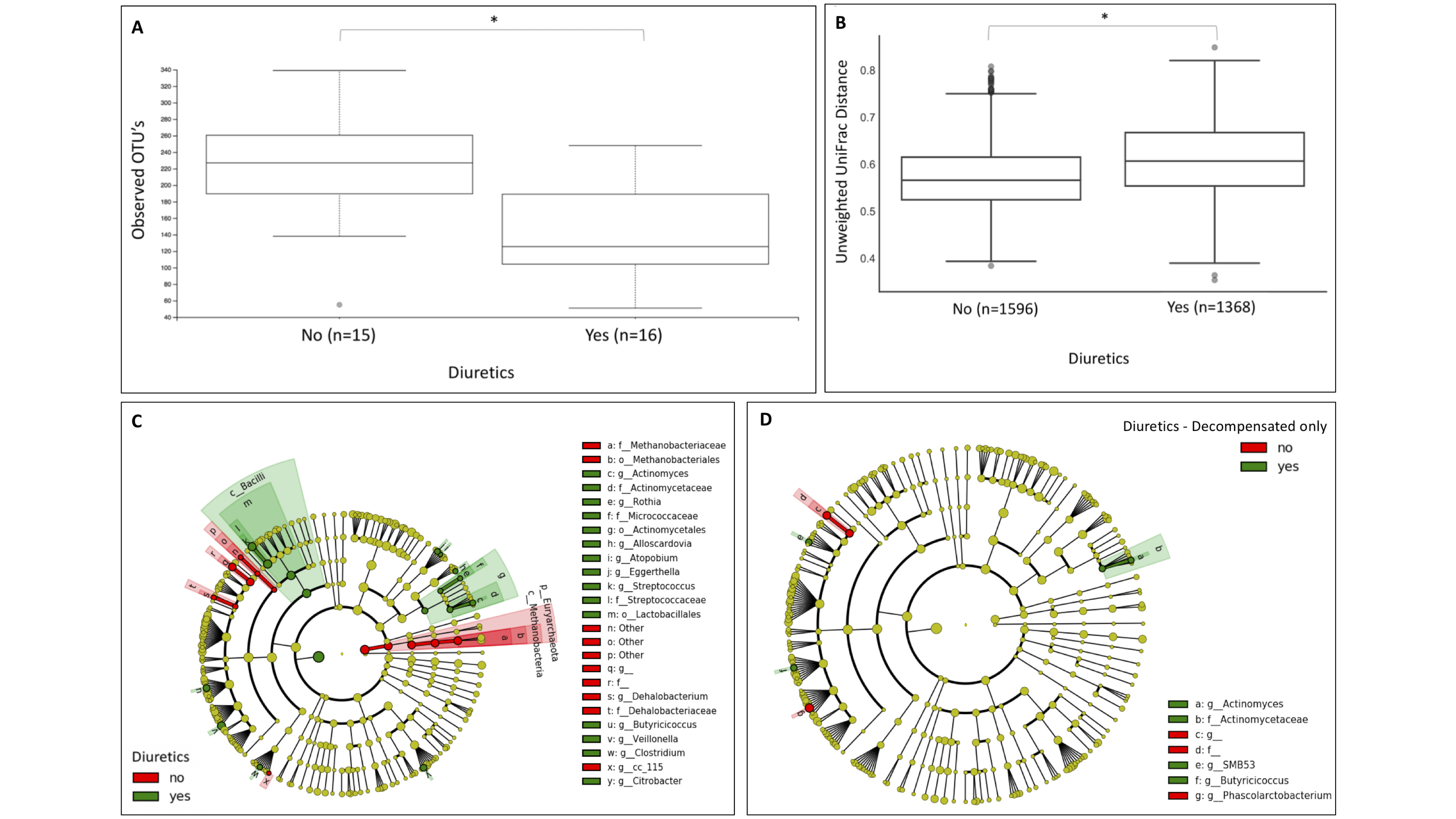

Supplement: FIG S3 [file mSystems.00153-20-sf003.tif]

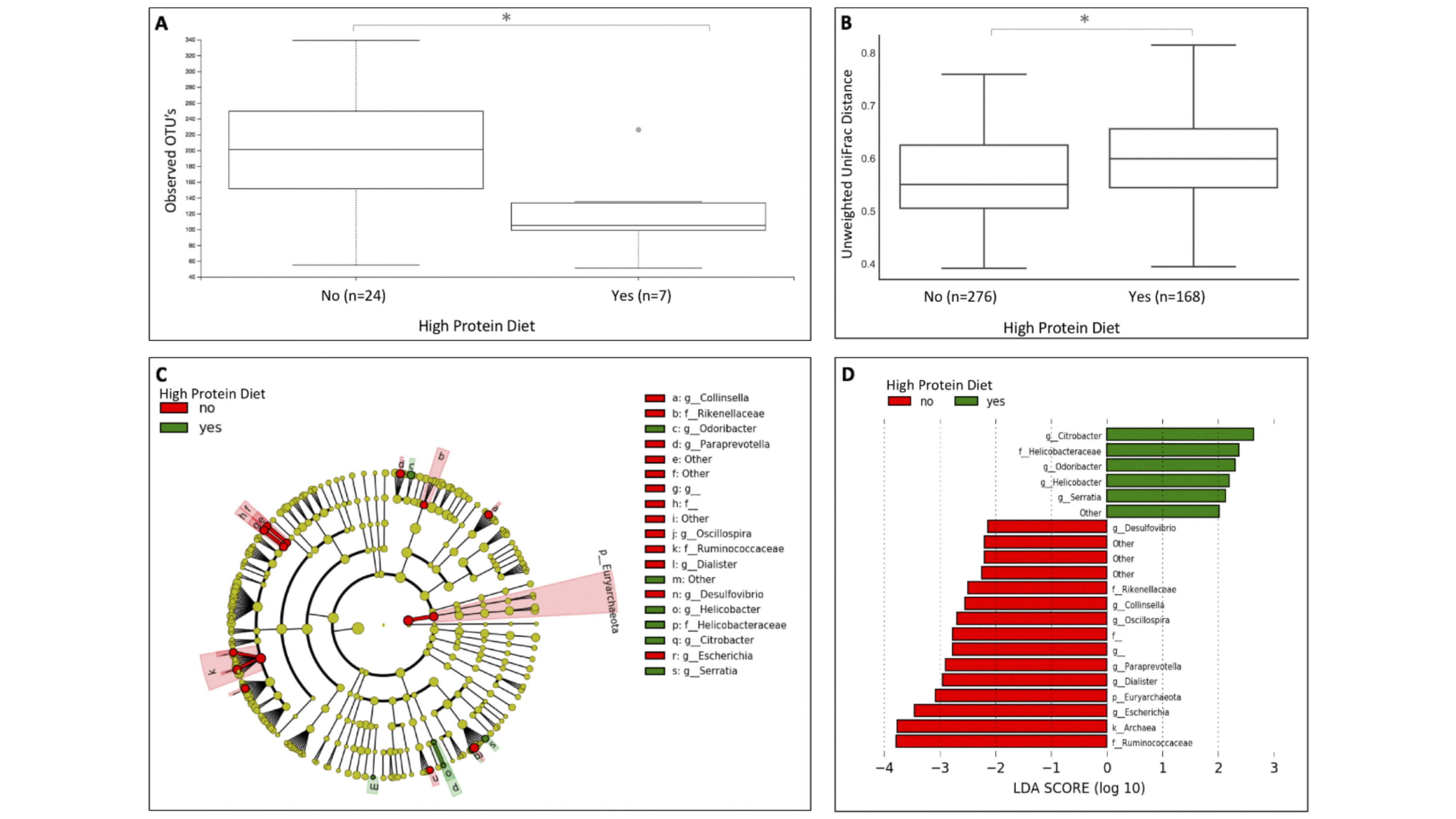

Supplement: FIG S4 [file mSystems.00153-20-sf004.tif]
