# Supplementary material for: Alterations in the Gut Microbiome in the Progression of Cirrhosis to Hepatocellular Carcinoma
Source: mSystems. 2020 Jun 16;5(3):e00153-20. doi: 10.1128/mSystems.00153-20 (PMC7300357; doi:10.1128/mSystems.00153-20)
Supplement: TABLE S1 [file mSystems.00153-20-st001.docx]

Table S1 - Study Questionnaire

| Date of sample collection:_________ Participant Code:_______ Doctor: ________ |
| --- |

**Questionnaire: Demographics information, lifestyle and nutrition habits and morbidity registration**

Questionnaire Completion Date: _______________

**Demographics information:**

**Last Name**: _____________ **First name**: _____________ **Date of birth**: _____________

**ID**: _____________ **Gender**: 1. Male 2. Female

**Marital status**: _____________ **Education**: _____________

**Occupation** (you can mark more than one option):

1. Office work that does not include public reception.
2. Office work that includes public Reception.
3. Patient care.
4. Working with children
5. Other _____________

Family background (Are there any known chronic diseases in the family):

____________________________________________________________

Weight (Kg): _____________ Height (cm): _____________

Duration of Liver Disease: _____________

**Nutritional care and counseling:** If you are adhering to a diet / nutritional regimen, specify the type of diet recommended and the degree of its application^*^ from 1-5. You can mark more than one option.

** Degree of diet application: 1. Not implementing the recommendations 2. Implementing a small part, rarely 3. Implementing some of the recommendations, not all the time 4. Implementing most of the recommendations, most of the time 5. Full implementation of the recommendations*

1. Weight loss Diet Degree of application: 1/2/3/4/5
2. High Protein Diet Degree of application: 1/2/3/4/5
3. Low Sodium Diet Degree of application: 1/2/3/4/5
4. Low Sugar Diet Degree of application: 1/2/3/4/5
5. Low Carbohydrates Diet Degree of application: 1/2/3/4/5
6. Low Fat Diet Degree of application: 1/2/3/4/5
7. Low Lactose Diet Degree of application: 1/2/3/4/5
8. Vegetarian Diet Degree of application: 1/2/3/4/5
9. Vegan Diet Degree of application: 1/2/3/4/5
10. Other: __________ Degree of application: 1/2/3/4/5

**Antibiotics Treatment**: Have you received Antibiotics in the last 3 months? If so, please specify the type of medicine and period of use: ______________________________

Did you experience any digestive system side effects (Diarrhea/ Constipation)? ______________________________

**Laxatives**: Have you received lactulose or other laxatives in the last 3 months? If so, please specify the type of medicine and period of use: _______________________

**Dietary Supplements**: Please specify any type of supplement that you use regularly:

1. ____________________ 2) ____________________
2. ____________________ 4) ____________________

**Food allergy/ Intolerance**: 1) No 2) Yes, please specify: ______________________

**Physical Activity (Weekly)**:

**Aerobic Activity (Running, Cycling, Swimming)**

1. Less than 30 minutes 2) 30-60 minutes 3) 60-90 minutes

4) 90-120 minutes 5) 120-150 minutes 6) More than 150 minutes

**Anaerobic Activity (Weight lifting, Resistance training, Yoga, Pilates)**

1. Less than 30 minutes 2) 30-60 minutes 3) 60-90 minutes

4) 90-120 minutes 5) 120-150 minutes 6) More than 150 minutes

**Please indicate the average weekly intake of the following foods:**

| **At least once a day** | **4-5 times a week** | **2-3 times a week** | **Once a week or less** | **Monthly/ Never** | **Food Type (Portion)** |
| --- | --- | --- | --- | --- | --- |
| 4 | 3 | 2 | 1 | 0 | Chicken/Turkey (100 gr) |
| 4 | 3 | 2 | 1 | 0 | Beef/pork (100 gr) |
| 4 | 3 | 2 | 1 | 0 | Egg (1 egg) |
| 4 | 3 | 2 | 1 | 0 | Fish and seafood (100 gr) |
| 4 | 3 | 2 | 1 | 0 | Leafy Greens (1 cup) |
| 4 | 3 | 2 | 1 | 0 | Fruit (1 large/ 2 small units) |
| 4 | 3 | 2 | 1 | 0 | Dairy Products |
| 4 | 3 | 2 | 1 | 0 | Artificial Sweeteners and "diet" products |
| 4 | 3 | 2 | 1 | 0 | Foods containing Saturated fats  (Processed meats, High fat cheeses, Butter) |
| 4 | 3 | 2 | 1 | 0 | Foods containing Unsaturated fats  (Olive oil, Avocado, Nuts) |
| 4 | 3 | 2 | 1 | 0 | Dietary Fiber (Whole wheat, Oats, Beans, Vegetables) |
| 4 | 3 | 2 | 1 | 0 | Alcoholic Beverages  (330 ml bear, 120 ml wine, 20 ml vodka) |
| 4 | 3 | 2 | 1 | 0 | Soda and Juices  (1 cup of 200 ml) |
| 4 | 3 | 2 | 1 | 0 | Foods containing High Fructose Corn Syrup (Salad Dressing, Candy, Breakfast cereal, Boxed dinners, chips, cookies and crackers) |

**Changes in eating habits:** Have you recently changed the eating frequency of one or more of the foods listed above? 1) No 2) Yes. Please specify: _____________
