# Supplementary material for: Alterations in the Gut Microbiome in the Progression of Cirrhosis to Hepatocellular Carcinoma
Source: mSystems. 2020 Jun 16;5(3):e00153-20. doi: 10.1128/mSystems.00153-20 (PMC7300357; doi:10.1128/mSystems.00153-20)
Supplement: TABLE S2 [file mSystems.00153-20-st002.docx]

**Table S2:** significantly altered bacteria in patients with cirrhosis as compared to healthy controls:

| **Feature** | **Class** | **Effect size** | **p value** |
| --- | --- | --- | --- |
| p__Proteobacteria | Cirrhosis | 3.338 | 0.032 |
| p__Proteobacteria.c__Gammaproteobacteria | Cirrhosis | 3.321 | 0.001 |
| c__Gammaproteobacteria.o__Enterobacteriales.f__Enterobacteriaceae | Cirrhosis | 3.309 | 0.002 |
| p__Proteobacteria.c__Gammaproteobacteria.o__Enterobacteriales | Cirrhosis | 3.309 | 0.002 |
| p__Verrucomicrobia.c__Verrucomicrobiae.o__Verrucomicrobiales | Cirrhosis | 2.857 | 0.043 |
| c__Verrucomicrobiae.o__Verrucomicrobiales.f__Verrucomicrobiaceae | Cirrhosis | 2.857 | 0.043 |
| o__Verrucomicrobiales.f__Verrucomicrobiaceae.g__Akkermansia | Cirrhosis | 2.857 | 0.043 |
| p__Verrucomicrobia.c__Verrucomicrobiae | Cirrhosis | 2.857 | 0.043 |
| p__Firmicutes.c__Clostridia.o__Clostridiales.f___Mogibacteriaceae_ | Cirrhosis | 2.515 | 0.037 |
| p__Firmicutes.c__Clostridia.o__Clostridiales.f___Mogibacteriaceae_.g__ | Cirrhosis | 2.513 | 0.026 |
| p__Firmicutes.c__Clostridia.o__Clostridiales.f__Clostridiaceae | Control | 3.095 | 0.000 |
| o__Bacteroidales.f___Paraprevotellaceae_.g__Paraprevotella | Control | 2.971 | 0.009 |
| p__Firmicutes.c__Clostridia.o__Clostridiales.f__.g__ | Control | 2.930 | 0.001 |
| p__Firmicutes.c__Clostridia.o__Clostridiales.f__ | Control | 2.930 | 0.001 |
| c__Clostridia.o__Clostridiales.f__Clostridiaceae.g__Clostridium | Control | 2.867 | 0.000 |
| o__Clostridiales.f__Lachnospiraceae.g__Lachnospira | Control | 2.730 | 0.047 |
| p__Actinobacteria.c__Coriobacteriia.o__Coriobacteriales | Control | 2.680 | 0.008 |
| p__Actinobacteria.c__Coriobacteriia | Control | 2.680 | 0.008 |
| c__Coriobacteriia.o__Coriobacteriales.f__Coriobacteriaceae | Control | 2.680 | 0.008 |
| c__Clostridia.o__Clostridiales.f__Clostridiaceae.g__SMB53 | Control | 2.586 | 0.009 |
| p__Firmicutes.c__Clostridia.Other | Control | 2.509 | 0.037 |
| c__Coriobacteriia.o__Coriobacteriales.f__Coriobacteriaceae.g__ | Control | 2.439 | 0.004 |
| o__Erysipelotrichales.f__Erysipelotrichaceae.g__RFN20 | Control | 2.348 | 0.015 |
| o__Coriobacteriales.f__Coriobacteriaceae.g__Collinsella | Control | 2.324 | 0.014 |
| p__Firmicutes.c__Clostridia.Other | Control | 2.292 | 0.037 |
| c__Bacteroidia.o__Bacteroidales.f__Rikenellaceae.g__ | Control | 2.212 | 0.009 |
| c__Opitutae.o___Cerasicoccales_.f___Cerasicoccaceae_.g__ | Control | 2.194 | 0.016 |
| c__Bacteroidia.o__Bacteroidales.f__Rikenellaceae | Control | 2.184 | 0.030 |
| c__Betaproteobacteria.o__Burkholderiales.f__Oxalobacteraceae | Control | 2.182 | 0.009 |
| c__Clostridia.o__Clostridiales.Other.Other | Control | 2.157 | 0.012 |
| p__Firmicutes.c__Clostridia.o__Clostridiales.Other | Control | 2.157 | 0.012 |
| o__Burkholderiales.f__Oxalobacteraceae.g__Oxalobacter | Control | 2.148 | 0.009 |
| o__Clostridiales.f__Ruminococcaceae.g__Butyricicoccus | Control | 2.115 | 0.001 |
| c__Opitutae.o___Cerasicoccales_.f___Cerasicoccaceae_ | Control | 2.065 | 0.016 |
| o__Clostridiales.f__Veillonellaceae.Other | Control | 2.055 | 0.037 |
| c__Clostridia.o__Clostridiales.f__Clostridiaceae.g__ | Control | 2.039 | 0.001 |
| p__Verrucomicrobia.c__Opitutae.o___Cerasicoccales_ | Control | 2.016 | 0.016 |
