# Supplementary material for: Alterations in the Gut Microbiome in the Progression of Cirrhosis to Hepatocellular Carcinoma
Source: mSystems. 2020 Jun 16;5(3):e00153-20. doi: 10.1128/mSystems.00153-20 (PMC7300357; doi:10.1128/mSystems.00153-20)
Supplement: TABLE S3 [file mSystems.00153-20-st003.docx]

**Table S3: Correlation Analysis of Food frequency questionnaire and gut bacteria in Cirrhosis group:**

| **Food item** | **Bacteria** | **Rho** | **p value** | **q value** |
| --- | --- | --- | --- | --- |
| Fish | o__Clostridiales.f__Ruminococcaceae.g__Anaerofilum | 0.528 | 0.001 | 0.220 |
| High protein products | o__Clostridiales.f__Veillonellaceae.g__Phascolarctobacterium | 0.500 | 0.001 | 0.220 |
| Fish | o__Desulfovibrionales.f__Desulfovibrionaceae.g__ | 0.437 | 0.006 | 0.294 |
| High sugar products | o__Enterobacteriales.f__Enterobacteriaceae.Other | -0.466 | 0.003 | 0.294 |
| Artificial Sweeteners | o__Rhodospirillales.f__Acetobacteraceae.g__Acetobacter | -0.391 | 0.015 | 0.392 |
| Fish | p__Tenericutes.c__Mollicutes.o__RF39.f__.g__ | 0.330 | 0.043 | 0.360 |
| Saturated fat | o__Verrucomicrobiales.f__Verrucomicrobiaceae.g__Akkermansia | 0.286 | 0.081 | 0.360 |
| Artificial Sweeteners | o__Verrucomicrobiales.f__Verrucomicrobiaceae.g__Akkermansia | 0.277 | 0.093 | 0.360 |

Spearman correlations analysis between parameters of food frequency questionnaires and fecal bacteria at the genus level in HCC-Cirrhosis group. In this table, the strongest correlations that received a p value >0.05 are presented. These correlations didn’t pass the multiple comparisons correction (q value<0.05).
