# Supplementary material for: Alterations in the Gut Microbiome in the Progression of Cirrhosis to Hepatocellular Carcinoma
Source: mSystems. 2020 Jun 16;5(3):e00153-20. doi: 10.1128/mSystems.00153-20 (PMC7300357; doi:10.1128/mSystems.00153-20)
Supplement: TABLE S4 [file mSystems.00153-20-st004.docx]

**Table S4:** Significantly altered bacterial composition in patients with HCC-Cirrhosis compared to healthy volunteers

| **Feature** | **Class** | **LDA effect size** | **p value** |
| --- | --- | --- | --- |
| p__Fusobacteria | HCC | 3.020 | 0.012 |
| p__Fusobacteria.c__Fusobacteriia | HCC | 2.726 | 0.012 |
| p__Fusobacteria.c__Fusobacteriia.o__Fusobacteriales | HCC | 2.653 | 0.012 |
| o__Fusobacteriales.f__Fusobacteriaceae.g__Fusobacterium | HCC | 2.852 | 0.012 |
| p__Proteobacteria.c__Gammaproteobacteria | HCC | 3.849 | 0.005 |
| c__Gammaproteobacteria.o__Enterobacteriales | HCC | 3.846 | 0.009 |
| o__Enterobacteriales.f__Enterobacteriaceae.g__Escherichia | HCC | 3.683 | 0.036 |
| o__Clostridiales.f__Veillonellaceae.g__Veillonella | HCC | 3.526 | 0.023 |
| o__Bifidobacteriales.f__Bifidobacteriaceae.g__Scardovia | HCC | 2.320 | 0.019 |
| p__Firmicutes.c__Clostridia.o__Clostridiales | Control | 3.411 | 0.019 |
| c__Clostridia.o__Clostridiales.f__Lachnospiraceae | Control | 3.289 | 0.045 |
| c__Clostridia.o__Clostridiales.f__Christensenellacea | Control | 2.641 | 0.026 |
| c__Erysipelotrichi.o__Erysipelotrichales.f__Erysipelotrichaceae | Control | 2.306 | 0.011 |
| c__Clostridia.o__Clostridiales.f__Clostridiaceae | Control | 2.266 | 0.008 |
| c__Clostridia.o__Clostridiales.f__Christensenellaceae | Control | 2.638 | 0.025 |
| o__Clostridiales.f__Lachnospiraceae.g__Lachnospira | Control | 3.196 | 0.040 |
| o__Clostridiales.f__Lachnospiraceae.g__Anaerostipes | Control | 2.714 | 0.005 |
| o__Clostridiales.f__Christensenellaceae.g__Christensenella | Control | 2.208 | 0.010 |
| o__Clostridiales.f__Ruminococcaceae.g__Ruminococcus | Control | 3.685 | 0.043 |
| o__Clostridiales.f__Ruminococcaceae.g__Butyricicoccus | Control | 2.762 | 0.000 |
| o__Clostridiales.f__Veillonellaceae.g__Mitsuokella | Control | 2.563 | 0.004 |
| c__Bacteroidia.o__Bacteroidales.f__Rikenellaceae | Control | 2.838 | 0.011 |
| o__Bacteroidales.f___Paraprevotellaceae_.g__Paraprevotella | Control | 3.548 | 0.027 |
| c__Coriobacteriia.o__Coriobacteriales.f__Coriobacteriaceae | Control | 2.941 | 0.022 |
| o__Coriobacteriales.f__Coriobacteriaceae.g__Adlercreutzia | Control | 2.159 | 0.001 |
| p__Verrucomicrobia.c__Opitutae | Control | 2.499 | 0.010 |
| p__Verrucomicrobia.c__Opitutae.o___Cerasicoccales_.f___Cerasicoccaceae_ | Control | 2.560 | 0.010 |
| p__Verrucomicrobia.c__Opitutae.o___Cerasicoccales_ | Control | 2.522 | 0.010 |
| p__Cyanobacteria | Control | 2.877 | 0.004 |
| p__Cyanobacteria.c__4C0d_2.o__YS2 | Control | 2.866 | 0.007 |
| p__Cyanobacteria.c__4C0d_2 | Control | 2.856 | 0.007 |
| p__Proteobacteria.c__Alphaproteobacteria | Control | 2.604 | 0.002 |
| p__Proteobacteria.c__Alphaproteobacteria.o__RF32 | Control | 2.640 | 0.000 |
| c__Betaproteobacteria.o__Burkholderiales.f__Oxalobacteraceae | Control | 2.596 | 0.035 |
| o__Burkholderiales.f__Oxalobacteraceae.g__Oxalobacter | Control | 2.579 | 0.035 |
