# Supplementary material for: Alterations in the Gut Microbiome in the Progression of Cirrhosis to Hepatocellular Carcinoma
Source: mSystems. 2020 Jun 16;5(3):e00153-20. doi: 10.1128/mSystems.00153-20 (PMC7300357; doi:10.1128/mSystems.00153-20)
Supplement: TABLE S5 [file mSystems.00153-20-st005.docx]

**Table S5:** **Feature importance analysis for random forest classifier between HCC and healthy controls:**

| Feature | numeric |
| --- | --- |
| o__Clostridiales; f__Veillonellaceae; g__Veillonella; s__dispar | 0.174 |
| o__Clostridiales; f__Ruminococcaceae; g__Faecalibacterium; s__prausnitzii | 0.146 |
| p__Firmicutes; c__Clostridia; o__Clostridiales | 0.142 |
| o__Clostridiales; f__Ruminococcaceae; g__Faecalibacterium; s__prausnitzii | 0.121 |
| c__Clostridia; o__Clostridiales; f__Ruminococcaceae | 0.119 |
| c__Bacteroidia; o__Bacteroidales; f__Bacteroidaceae; g__Bacteroides; s__ | 0.105 |
| o__Clostridiales; f__Lachnospiraceae; g__[Ruminococcus]; s__gnavus | 0.101 |
| o__Clostridiales; f__Ruminococcaceae; g__Ruminococcus; s__ | 0.0912 |
