# Supplementary material for: Alterations in the Gut Microbiome in the Progression of Cirrhosis to Hepatocellular Carcinoma
Source: mSystems. 2020 Jun 16;5(3):e00153-20. doi: 10.1128/mSystems.00153-20 (PMC7300357; doi:10.1128/mSystems.00153-20)
Supplement: TABLE S6 [file mSystems.00153-20-st006.docx]

**Table S6: Correlation Analysis of Food frequency questionnaires and gut bacteria in HCC-Cirrhosis group:**

| FFQ reported consumption | Bacteria | Rho | p value | q value |
| --- | --- | --- | --- | --- |
| Artificial Sweeteners | o__Verrucomicrobiales.f__Verrucomicrobiaceae.g__Akkermansia | 0.610 | 0.002 | 0.021 |
| High sugar foods | o__Synergistales.f__Synergistaceae.g__Cloacibacillus | 0.584 | 0.003 | 0.021 |
| High saturated fat foods | o__Coriobacteriales.f__Coriobacteriaceae.g__Adlercreutzia | -0.501 | 0.013 | 0.354 |
| Fish | o__Actinomycetales.f__Actinomycetaceae.g__Actinomyces | -0.475 | 0.019 | 0.354 |
| High protein foods | o__Actinomycetales.f__Micrococcaceae.g__Rothia | -0.462 | 0.023 | 0.354 |
| High fiber foods | o__Enterobacteriales.f__Enterobacteriaceae.g__Klebsiella | 0.472 | 0.020 | 0.641 |
| High fiber foods | o__Desulfovibrionales.f__Desulfovibrionaceae.g__ | 0.468 | 0.021 | 0.641 |
| Dairy | o__Pasteurellales.f__Pasteurellaceae.g__Haemophilus | -0.456 | 0.025 | 0.641 |
| High protein Foods | o__Enterobacteriales.f__Enterobacteriaceae.g__Klebsiella | -0.418 | 0.042 | 0.808 |

Spearman correlations analysis between parameters of food frequency questionnaires and fecal bacteria at the genus level in HCC-Cirrhosis group. In this table, the strongest correlations that received a p value >0.05 are presented. Two of these correlations passed the multiple comparisons correction (q value<0.05) – Artificial sweeteners consumption was significantly correlated with Verrucomicrobia genus *Akkermansia muciniphila*  and consumption of product containing high amounts of sugar with Synergistetes genus *Cloacibacillus* .
